# Characteristics of Children’s Media Use and Gains in Language and Literacy Skills

**DOI:** 10.3389/fpsyg.2020.02224

**Published:** 2020-09-09

**Authors:** Rebecca A. Dore, Jessica Logan, Tzu-Jung Lin, Kelly M. Purtell, Laura Justice

**Affiliations:** ^1^Crane Center for Early Childhood Research and Policy, The Ohio State University, Columbus, OH, United States; ^2^Department of Educational Studies, The Ohio State University, Columbus, OH, United States; ^3^Department of Human Sciences, The Ohio State University, Columbus, OH, United States

**Keywords:** media, language, literacy, co-viewing, joint media engagement, interactivity

## Abstract

Media use could be detrimental to children’s language and literacy skills because it may displace other language-enhancing activities like shared reading and caregiver-child interactions. Furthermore, the extent to which children use media with adults (joint media engagement), the extent to which they use interactive media (apps/games), and the time of the day and week during which media use occurs may attenuate any negative effects. The current study examines the relation between characteristics of children’s media use and gains in first graders’ language and literacy skills. Children (*N* = 488) completed direct assessments of language and literacy skills in the spring of kindergarten and the spring of first grade. Parents reported how many hours children used both interactive and non-interactive media during different times of the day on the most recent weekday and weekend day and responded to items about the extent to which they engage with their children during media use. A quadratic relationship between media use and language gains showed that a moderate amount of media use was related to larger language gains, whereas high use was related to smaller gains. For literacy, an interaction between media use and joint media engagement showed a small negative effect of media use at low levels of joint media engagement and little to no relation between media use and literacy gains at higher levels of joint media engagement. Children’s language and literacy skills were not predicted by either the proportion of media time that was spent with apps/games or morning and weekday media use. These results show that moderate amounts of media use may not be a negative influence on children’s developing language skills, whereas high levels may displace other language-enhancing activities. Additionally, joint media engagement may play an important buffering role in the relation between media use and early literacy skills, aligned with current recommendations encouraging co-viewing.

## Introduction

Popular press coverage often highlights studies showing associations between how many hours of “screen time” children are exposed to and negative outcomes, potentially fueling concerns among parents and caregivers about their children’s use of media and technology. One domain that has been investigated in prior research is the relation between media use and language development. Indeed, several studies have found that media exposure during toddlerhood or preschool is associated with lower language development in subsequent years. Clarke and Kurtz-Costes (1997) found that preschoolers’ TV viewing was negatively associated with several domains of school readiness. Similarly, Pagani et al. (2013) found that every additional hour that children watched TV at 29 months of age was associated with 11% lower vocabulary scores at 65 months of age. However, findings are inconsistent, with other research finding no association between media exposure and language development. Patterson (2002) examined expressive vocabulary size and television watching among 21‐ to 27-month old bilingual toddlers and found that TV watching was not associated with vocabulary size in either language. Schmidt et al. (2009) found similar results for TV viewing at 6 months, 1 year, and 2 years and vocabulary skills at 3 years. More recently, Taylor et al. (2017) reported on an upper-socioeconomic status (SES) sample of children in the United Kingdom and found that TV and mobile device use was not predictive of children’s vocabulary skills for children between 6 and 36 months of age. Notably, in both of these studies time spent being read to was associated with language (although only for 6‐ to 18-month-olds in Taylor et al.), suggesting that the variability in children’s vocabulary scores was meaningful and associated with other characteristics of the home environment. Together, the only consistent finding in this literature is inconsistency, suggesting that studies may be leaving out critical factors that may help explain discordant findings; some of these factors are speculated on below and tested in the current research. This represents a critical gap in our understanding and precludes the development of evidence-informed recommendations.

In the current study, we investigate four hypotheses that might explain these mixed findings. First, we assess the possibility that there are nonlinear relations between media use and children’s skill gains. Our prior research has demonstrated that any (weak) relations between media use and language development are best represented as a threshold effect rather than a straightforward linear relation, such that increases from small to moderate amounts of media use are not related to children’s skill gains, whereas larger amounts of media use are related to lower gains (Dore et al., in press). Studies testing only for linear relations may miss meaningful associations that manifest as quadratic relations. Here, we use a continuous measure of children’s media use that should be more sensitive to potential associations and test both linear and quadratic relations to uncover possible associations with children’s skill gains.

A second hypothesis that might explain mixed findings in this domain is that media use has differential effects on language and literacy development depending on the extent to which it disrupts other beneficial activities. This idea is grounded in Vygotsky’s theory of language development, highlighting the idea that language acquisition is embedded in social interaction and that talk that is contingent and responsive to children’s verbalizations and actions should support language development (Vygotsky, 1962; Bruner, 1983). Thus, time spent with media could be detrimental to the children’s language skills because it may *displace* language-enhancing activities. For example, Vandewater et al. (2006) found that time spent watching TV was negatively related to time spent with parents and siblings, as well as creative play.

Following this research, media use may have a negative effect on language growth only to the extent that it inhibits caregiver-child interaction and caregiver language input. In other words, joint media engagement may moderate the association between children’s media use and language skills. Joint media engagement refers to experiences in which caregivers and children use the same media at the same time, are involved in the content together, and are prompted by what they are seeing to interact with each other and bring more meaning to what they are watching or doing (Stevens and Penuel, 2010; Takeuchi and Stevens, 2011; Guernsey and Levine, 2015; see Dore and Zimmermann, 2020, for a review). When parent-child joint media engagement is frequent, children’s development may be more positive because the media experience does not replace contingent caregiver-child interaction but instead extends it to a new context. Some research has found that the negative association between preschoolers’ television exposure and a standardized measure of language development is entirely explained by accounting for adult-child conversations, suggesting that joint media engagement may influence language (Zimmerman et al., 2009). Indeed, there is no relation between infants’ media exposure (television, videos/DVDs, movies, and games) and a standardized measure of language development when caregivers report frequent joint media engagement (Mendelsohn et al., 2010). Additionally, Strouse et al. (2013) found that children understood a story and learned new words better when their parents were trained to use joint media engagement while viewing an educational video by pausing and asking their child questions about the content. Thus, to the extent that caregivers use media with their children and engage in conversation around media, any negative effects on language development may be attenuated. Joint media engagement is variable across families (Connell et al., 2015) and is thus a possible hidden moderator of media on language trajectories. Thus, we predict that joint media engagement will moderate the association between media use and children’s language gains, such that any negative association between media use and language will be attenuated when joint media engagement is high.

Notably, although considerable research has investigated the role of media in young children’s language development, less focus has been placed on early literacy skills. This is critical, because early literacy skills are a major predictor of later reading performance (National Early Literacy Panel, 2008). Indeed, language and literacy skills are intricately related during the early school years and work together to influence reading ability (Snow, 1991; Snow et al., 1995; Torppa et al., 2010). As with language development, the displacement hypothesis suggests that media use may take the place of activities like shared storybook reading, which are linked to the development of children’s early literacy skills. Indeed, Khan et al. (2017) found that children’s TV viewing was negatively related to the frequency of parent-child reading. However, any relation between media use and literacy development may also be moderated by joint media engagement, as adults can support children’s literacy learning from educational TV when they scaffold the interaction by asking children questions and providing feedback (e.g., Reiser et al., 1984). Recent research by Hutton et al. (2020) also supports an association between media use and literacy skills. The researchers created a new composite measure designed to align with the American Academy of Pediatrics’ recommendations for young children’s media use. The parent report measure contained 15 items assessing access to screens, frequency of use, media content, and caregiver-child co-viewing (akin to joint media engagement). Parents of preschoolers completed the measure and children complete a standardized measure of core emergent literacy skills. Results showed that the media measure was negatively related to emergent literacy skills, although the composite nature of the assessment makes it impossible to determine the specific role of joint media engagement as opposed to other aspects of children’s media use (i.e., quantity and content). A more nuanced understanding of how both the quantity of children’s media use and joint media engagement relate to both language and literacy skills will provide a broader lens through which to consider the role of media in child development. As with language skills, we hypothesize that there may be a negative, quadratic relation between media use and literacy gains and a moderating effect of joint media engagement, such that any negative association between media use and literacy will be attenuated when joint media engagement is high.

A third hypothesis to explain mixed findings related to the effects of media use on children’s language and literacy skills is the extent to which the media is interactive. Digital games and apps may be more supportive of language and literacy development than non-interactive media use, as they are interactive and responsive to the child’s actions in a way that a television show is not (Sheehan and Uttal, 2016). Indeed, existing research focuses primarily on television use, whereas an increasing amount of children’s media use comes from interactive media like apps and games on mobile devices. It is possible that children learn better from touchscreens, as learning is enhanced when children are actively engaged in an activity (Hirsh-Pasek et al., 2015). Joint attention and serve-and-return interactions are important for word learning (Tomasello and Farrar, 1986; Bloom et al., 1987) and apps mimic some of those features – for example, by providing labels immediately after children touch an object or responding to incorrect responses with an appropriate hint.

However, mixed findings emerge when this idea is tested empirically. Some research finds that preschoolers readily learn new information from apps on touchscreen devices (Huber et al., 2016) and that toddlers who use more interactive media (apps/games) learn new information better from media in general, suggesting that experiences with interactivity may have shown them that media can be responsive and a reliable source of information (Kirkorian and Choi, 2016). Yet, other studies show that preschoolers learned less from playing an interactive game than when passively watching a video of gameplay (Aladé et al., 2016; Schroeder and Kirkorian, 2016) or that the effect of interactivity depends on children’s age or sex (Choi and Kirkorian, 2016; Kirkorian et al., 2016; Russo-Johnson et al., 2017). These studies have primarily focused on lab-based learning tasks (e.g., finding the location of a hidden object) and little research to our knowledge has examined how media interactivity relates to language and literacy development. We hypothesize that media interactivity will moderate the association between media use and language and literacy development, such that any negative association between media use and language and literacy will be attenuated when media interactivity is high.

A fourth hypothesis is that the time of the day and week during which media use occurs could influence the relation between media use and language development. Recent studies have suggested that fantastical television (Lillard et al., 2015) and noneducational cartoons (Huber et al., 2018) may inhibit children’s executive function skills and if children use these media immediately prior to school, it may disrupt opportunities for learning. Furthermore, following from the displacement hypothesis, the types of activities that are displaced by media use may differ for weekdays and weekends, such that more language-enhancing activities are displaced during the week, whereas weekend media use may be likely to displace less constructive activities. If true, these hypotheses would suggest that when more of children’s media use occurs in the morning before school and on the weekdays, language and literacy development may be more negatively affected than when media use occurs during other times the day and week.

We focus on children transitioning from kindergarten to first grade because research on media and language has focused primarily on children under 3 years of age (see Linebarger and Vaala, 2010, for a review) and there is relatively less evidence for the role of media in language and literacy development among older children. Children in this age range are gaining more advanced vocabulary and language skills, as well as beginning to learn to read and gain important early literacy skills (Farkas and Beron, 2004). It is vitally important to understand predictors of these skills among children during the early elementary years, given the role of these skills in predicting reading achievement (e.g., Blachman, 1984). Media use is also higher in this age range than during early childhood (Rideout, 2017), perhaps partially because of less restrictive recommendations from the American Academy of Pediatrics for older children (AAP Council on Communications and Media, 2016). Thus, this period may be an ideal time for interventions to reduce media use or influence its content and context. Understanding the role of media use in development for children in this age range is important to inform future developmentally-specific recommendations.

Importantly, we measure and control for several demographic factors that may be related to both media use (or characteristics of media use) and language and literacy gains, as relations between media and children’s outcomes are often attenuated by including proper control variables (e.g., Orben and Przbylski, 2019). By controlling for these variables, we will have greater confidence that any relations between media use and language and literacy development are unique and meaningful associations.

In all, the current study addresses four research questions: (1) To what extent is the quantity of children’s media use associated with gains in the language and literacy skills of children from kindergarten to first grade? *We hypothesize that there will be quadradic, rather than linear, associations between media use and language and literacy skills, such that media use is only negatively associated with skill gains at high levels*. (2) To what extent does the degree of joint media engagement moderate the association between the quantity of media use and gains in language and literacy skills? *We hypothesize that joint media engagement will moderate the association between media use and children’s language and literacy gains, such that any negative association between media use and language and literacy will be attenuated when joint media engagement is high*. (3) To what extent does the interactivity of the media moderate the association between the quantity of media use and gains in language and literacy skills? *We hypothesize that media interactivity will moderate the association between media use and children’s language and literacy gains, such that any negative association between media use and language and literacy will be attenuated when media interactivity is high*. (4) To what extent is morning and weekday media use associated with gains in language and literacy skills? *We hypothesize that when more of children’s media use occurs in the morning before school and on the weekdays, language and literacy gains may be smaller than when media use occurs during other times the day and week*.

## Materials and Methods

### Participants

Participating teachers in a large school district in Ohio received financial incentives as part of the larger study and all children in their classrooms were recruited. Of those asked to participate in preschool, 64.5% consented. Data from the spring of kindergarten and the spring of first grade year are reported in the current study^1^.

Of the children whose parents consented for them to participate, approximately 55.4% of families (representing 488 children) responded to the survey items about child media use to be included in the current analysis^2^. Thus, data from 488 children (53.2% males) primarily between 6 and 8 years of age (*M* = 84.9, *SD* = 4.4) are included. See Table 1 for sample demographics.

**Table 1:** Descriptive statistics for all study variables.

Continuous variables	Mean	*SD*
WJ Picture Vocabulary (K)	473.8	10.2
WJ Picture Vocabulary (first)	480.4	9.7
WJ Letter-Word Identification (K)	401.8	30.1
WJ Letter-Word Identification (first)	446.1	30.8
Weekly media use in hours	23.5	13.2
Joint media engagement score	26.8	5.9
**Factors**	**Percentage (%)**	
Mother’s education		
Less than high school diploma	11.3	
High school diploma or GED	41.4	
Associate’s degree	16.0	
Bachelor’s degree	21.4	
Graduate or professional degree	9.9	
Number of adults in the home		
One	11.1	
Two	73.6	
More than two	15.2	
Child’s race		
White	74.1	
Hispanic or Latino	14.3	
Black or African-American	4.0	
Asian	3.1	
Multiple races	10.4	
Other	8.4	

### Procedures

We used two time points from the larger longitudinal project to address our research questions: the spring of kindergarten and the spring of first grade. Children’s language and literacy skills were directly assessed in the spring of kindergarten and the spring of first grade. In the spring of first grade only, caregivers reported on children’s media use, as well as other child and family demographics characteristics.

#### Quantity of Child Media Use

Parents were asked how long their child spent using two types of media (“Watching any kind of video including TV, movies or short clips on any type of device” and “Using apps or games on any type of electronic device”) during three different time periods (the most recent weekday before school, the most recent weekday after school, and the most recent weekend day). For each time period, there were eight response options from “None” to “More than 3 h” with intervening options in half an hour increments. To create a total weekly media score, any response of “More than 3 h” was coded as 4 h and these items were aggregated by multiplying the weekday score by 5 and the weekend score by 2 and summing. Outliers were winsorized by replacing values that were more than three *SD*s above the mean with that value; 1.6% of the data were replaced in this manner.

#### Joint Media Engagement

To assess joint media engagement, we created a new measure informed by existing scales of caregiver mediation based on particular content (e.g., Rasmussen et al., 2016), focused exclusively on television viewing (e.g., Valkenburg et al., 1999; Nathanson et al., 2013), or exclusively examining co-use with children (e.g., Rideout, 2017). Thus, our measure assesses the extent to which adults use media with the child and the extent to which adults talk to the child about media. Responses are on a 6-point scale from strongly disagree to strongly agree.

Confirmatory factor analyses revealed that three items did not load with the rest of the items in the scale and were removed for analyses. All other items loaded at 0.438 or above, comparative fit index (CFI) = 0.852, root mean square error of approximation (RMSEA) = 0.169, χ^2^ < 0.001, and standardized root mean square residual (SRMR) = 0.088. Of the final items, one asks about co-viewing, one asks about distracted co-viewing (caregiver is in the room but engaged in another task), three ask about conversation during media use (two reverse scored), and two ask about discussing media after use. These seven items were summed to create a joint media engagement score. See Table 2 for the final items.

**Table 2:** Joint media engagement items.

Item
It is usually in the same room as me or another adult. I am not sure whether they are watching videos or using apps/games.^*^ I comment on or ask my child questions about what is happening. I do not interrupt him/her to talk about what he/she is doing or watching.^*^ We do not talk much about what he/she is doing or watching.^*^ I bring up what he/she saw or did in other conversations. We talk about it beforehand.

* Reverse scored.

#### Interactivity of the Media

The quantity items described above were used to create a variable representing how much children use interactive (using apps/games regardless of device) vs. non-interactive media (watching video and regardless of device). Specifically, we created proportion scores by dividing the time children spent with apps/games by their total media time.

#### Language Skills

To assess language skills, children completed the Picture Vocabulary subtest of the *Woodcock Johnson Test of Achievement-III* (WJ-III; Woodcock et al., 2007) in the spring of kindergarten and the spring of first grade. The initial items of the subtest require children to choose the picture that fits the named word for the initial items, and then later items require children to provide names for each picture (44 items total). Six consecutive correct items are needed to establish test basal and six consecutive incorrect responses terminate the test. Reliability was adequate (0.80) and W-scores were used to examine student growth.

#### Literacy Skills

To assess literacy skills, children completed the Letter-Word Identification subtest of the *Woodcock Johnson Test of Achievement-III* (WJ-III; Woodcock et al., 2007) in the spring of kindergarten and the spring of first grade. This subtest (76 items total) requires children to identify individual letters and then read individual words of increasing difficulty. Six consecutive correct items are needed to establish test basal and six consecutive incorrect responses terminate the test. Reliability was adequate (0.94) and W-scores were used to examine student growth.

## Results

### Preliminary Analyses

We first report descriptive statistics related to children’s media use. According to parent report, children used media for a mean of 23.5 h per week (*SD* = 13.2) or over 3 h per day (*M* = 3.36).

On our scale for joint media engagement, responses could range from 0 to 35, with higher scores representing more joint media engagement. The mean joint media engagement score total was 26.8 (*SD* = 6.01), *N* = 467. Although the distribution was negatively skewed, scores from 0 to 35 were represented in the data.

The mean media interactivity proportion was 40.5% (*SD* = 17.1), suggesting more video watching than app/game use. These scores ranged from 0 to 1, indicating that some children used all videos and no apps/games, whereas other children used all apps/games and no video.

In relation to time of day and week, 59.8% of children were reported to use media before school in the morning and 97.7% were reported to use media on weekdays after school. On average, children used media for almost 1 h before school in the morning (*M* = 0.95, *SD* = 1.36) and over 3 h on weekdays (*M* = 3.11, *SD* = 2.30). Parents reported that 99.0% of children used media on the most recent weekend day.

### Association Between the Quantity of Media Use and Children’s Language and Literacy Gains

To address our first research question, we conducted multilevel regression models accounting for classroom variance. To assess changes in children’s language and literacy skills, children’s first-grade scores were dependent variables and the models controlled for kindergarten scores in both language and literacy. The models also controlled for age, gender, race, mother’s education, and number of adults in the household, as these may be related to both media use and language and literacy gains.

**Figure 1:**
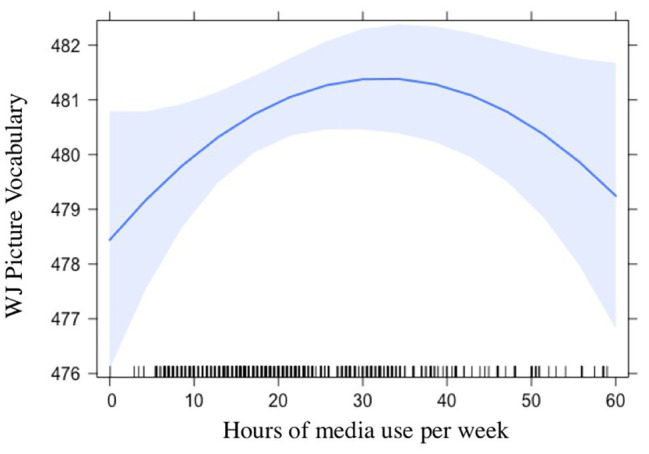
Association between weekly media use and language gains. Each vertical line along the X-axis represents one child.

For language skills, media use did not predict gains in the linear model (*p* = 0.33). However, results showed a quadratic relation (*B* = −13.9, *p* = 0.03) showing that children who use a moderate amount of media have the largest language gains, whereas both the lowest and the highest levels of media use are associated with smaller language gains, see Table 3; Figure 1. For the models predicting literacy, neither linear nor quadratic effects were significant (*ps* > 0.35).

**Table 3:** Predicting language gains: results of multilevel regression model (*N* = 419).

Predictor	*B*	*SE*	*p*
Intercept	171.2	16.0	<0.0001^***^
Baseline language	0.60	0.04	<0.0001^***^
Baseline literacy	0.04	0.01	0.0001^**^
Media use	6.33	6.63	0.34
Media use^2^ (quadratic)	−13.94	6.57	0.03^*^
Gender	−0.08	0.64	0.90
Age	0.07	0.07	0.32
Race (White)	2.81	0.96	0.004^**^
Mother’s education	0.01	0.29	0.96
Number of adults in household	−0.80	0.50	0.11

Outcome is Picture Vocabulary W-scores.

* *p* < 0.05;

** *p* < 0.01;

*** *p* < 0.001.

### Joint Media Engagement as a Moderator of Associations Between Media Use and Children’s Language and Literacy Gains

To address our second research question, we conducted models adding joint media engagement as a moderator of the association between media quantity and children’s skill gains.

First, we examined whether joint media engagement moderated the relation between media use and skill gains. When predicting language, the interaction was not significant in either the linear or the quadratic models (*ps* > 0.23).

**Figure 2:**
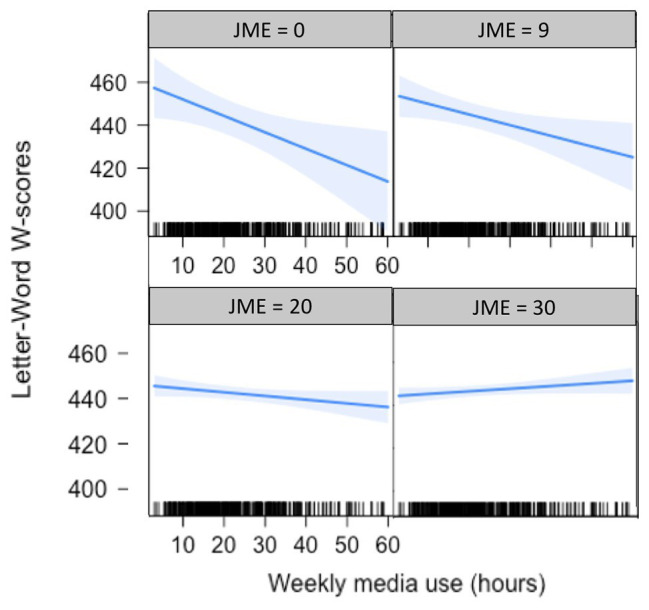
Association between weekly media use and literacy gains, moderated by joint media engagement (JME). Each panel represents a different level of joint media engagement score, starting from a low score of 0 on the top left, going to a high score of 30 on the bottom right.

For the models predicting literacy, there was a significant interaction between media use and joint media engagement (*B* = 0.03, *p* = 0.02), showing a small negative effect of media use at low levels of joint media engagement and little to no relation between media use and literacy gains at higher levels of joint media engagement, see Table 4; Figure 2.

**Table 4:** Predicting literacy gains: results of multilevel regression model (*N* = 419).

Predictor	*B*	*SE*	*p*
Intercept	119.96	48.2	0.01^*^
Baseline literacy	0.77	0.03	<0.0001^***^
Baseline language	0.07	0.11	0.55
Media use	−0.66	0.33	0.05^*^
Gender	1.50	1.79	0.40
Age	−0.11	0.20	0.59
Race (White)	1.22	2.94	0.68
Mother’s education	2.29	0.84	0.007^**^
Number of adults in household	−0.82	1.4	0.56
Media use × Joint media engagement	0.03	0.01	0.02^*^

Outcome is Letter-Word W-scores.

* *p* < 0.05;

** *p* < 0.01;

*** *p* < 0.001.

### Interactivity of the Media as a Moderator of Associations Between Media Use and Children’s Language and Literacy Gains

To address our third research question, we conducted models adding the proportion of children’s media use that interactive media (apps/games) as a moderator of the association between media quantity and children’s skill gains. There were no interactions between media use and the proportion of children’s interactive media use for either language or literacy (*p*s > 0.48).

### Association of Morning and Weekday Media Use and Children’s Language and Literacy Gains

To address our fourth research question, we conducted models parallel to those for research question one but predicting language and literacy gains from the quantity of children’s morning media and, separately, from quantity of children’s weekday media use. There were not significant associations between morning (*p*s > 0.13) and weekday (*p*s > 0.54) media use for either language or literacy skills.

## Discussion

These results shed light on media use and children’s language and literacy skills. We found that for language, the effect of media use differed by the level of use: children who used a moderate amount of media had the largest language gains, whereas both the lowest and the highest levels of media use are associated with smaller language gains. Our results for literacy showed that the association with media use depended on joint media engagement, such that when joint media engagement was low, media use was negatively related to literacy gains, but at high levels of joint engagement, this relation was not present. However, counter to our predictions, the relation between media use and language was not moderated by joint media engagement, and there was no main effect of media use on literacy gains. Furthermore, interactivity of the media and morning and weekday media use were not associated with either language or literacy gains.

Descriptively, our results showed that children used over 3 h of media per day on average. This level of usage is in line with a previous nationally representative survey showing that children between five and eight used almost 3 h of media per day on average (Rideout, 2017). The slightly higher use in our sample may be due to several factors, including our more nuanced methodology for asking about media use during different times of day.

Our first research question focused on the association between media use and children’s language and literacy skill gains. The quadratic relation between media quantity and language skills runs counter to the idea that any amount of media use is detrimental for development. Our results showed that children who had a moderate amount of weekly media use were likely to have higher language gains than children who had no or very little weekly media use. This finding may reflect the potential educational value of some programs (e.g., Mares and Pan, 2013) or the idea that media can expose children to new vocabulary and concepts in a similar way to children’s picture books (Lavigne et al., 2015; Montag et al., 2015). However, at higher levels, increased media use had a negative relation with children’s language gains, which is in line with social interaction theories of language development (Vygotsky, 1962; Bruner, 1983) and prior suggestions that media use can replace other valuable language-enhancing activities (e.g., Vandewater et al., 2006; Khan et al., 2017). This finding is an important extension of prior research in this domain, which has demonstrated mixed results with some studies finding negative linear associations between media use and children’s language skills (e.g., Clarke and Kurtz-Costes, 1997; Pagani et al., 2013) whereas others find no relation (e.g., Patterson, 2002; Schmidt et al., 2009; Taylor et al., 2017). By ignoring potential quadratic relations, these prior studies may be missing a meaningful association, potentially explaining conflicting findings.

Literacy gains, on the other hand, appeared to differ based on the extent to which caregivers reported engaging with children during media use. When joint media engagement was low, media use was negatively related to literacy gains, perhaps because it replaced activities that are more likely to focus on literacy skills, like shared reading. However, at high levels of joint engagement, this relation was non-significant, perhaps because parents who use media with children are likely to use opportunities within media to provide practice with literacy skills, in line with research suggesting that such joint engagement can support children’s learning (Reiser et al., 1984; Strouse et al., 2013). One might expect that joint media engagement is acting as a proxy for general parenting quality or home environment, such that any association is not due to media use specifically but instead shows that higher quality parenting or home environment is associated with literacy gains. However, in a supplementary analysis, we found no main effect of joint media engagement on literacy gains, suggesting that an association between joint media engagement and general parenting quality or home environment outside of media use is unlikely to explain the relation.

These contrasting results beg the question of why our first hypothesis was not supported for literacy (i.e., there was no main effect of media use on literacy gains) and our second hypothesis was not supported for language (i.e., joint media engagement did not moderate the association between media use and children’s language gains). Although these disparate findings were not predicted, we have several speculations that may explain these results. First, our measure of joint media engagement was positively skewed, with most caregivers reporting engaging in these behaviors at moderate to high levels. For these children, there was little to no relation between media use and literacy gains. Thus, a paucity of data points at the low end of the distribution, where an association does emerge, may have precluded our ability to detect a main effect of media use on gains in children’s literacy skills across the year. Second, there is more growth in literacy skills than in language skills across first grade, meaning that there was less variability to predict in language skills and thus, lower power to see a potential moderation effect even though the main effect emerged. However, it is also possible that this finding represents a true null effect, indicating that, counter to prior research with infants and preschoolers (Zimmerman et al., 2009; Mendelsohn et al., 2010), joint media engagement does not support language development for children during the early elementary years, perhaps because there are additional influences on these skills that contribute more variance (e.g., school and peers). Future research would do well to investigate skill gains over longer developmental periods and attempt to develop more sensitive measures of joint media engagement.

It was also somewhat surprising that children’s language and literacy skills were not predicted by the proportion of media time that was spent with apps/games, the indicator of interactivity of the media in this study. This finding runs counter to theoretical approaches suggesting that children may learn more from interactive media (Sheehan and Uttal, 2016) and ideas in the popular press that interactive screen time may be more beneficial for development than video. However, at least for overall media exposure and for language and literacy skills, interactive and non-interactive media seem to have similar relationships with skill gains. Results may differ for educational media (Hirsh-Pasek et al., 2015) or for apps and games that include developmentally appropriate guidance, like scaffolded feedback (Callaghan and Reich, 2018), which better mimic the serve-and-return interactions that are important for language learning (Bloom et al., 1987).

Similarly, there was no relation between children’s media use in the mornings before school or on weekdays and children’s language and literacy gains. Although prior research has shown that certain types of media can have immediate impacts on executive function skills (Lillard et al., 2015; Huber et al., 2018), which may disrupt learning, our data does not allow us to determine what types of media children were using in the morning before school or what academic content (i.e., literacy, math, etc.) they were exposed to immediately upon their arrival at school. This finding, in combination with the finding that weekday media use was not differentially associated with children’s skill gains, suggests that it is the overall quantity of children’s media use that is related to language and literacy skills, not use at any specific time of the day or week.

Our primary findings have several important implications for media use among young children. First, they suggest that moderate amounts of media use are not detrimental, and may even be beneficial for language growth, at least by the first grade year. Although this finding does not indicate that increasing media use should be recommended over established language-promoting activities like book reading, it does suggest that when left to their own devices, families who limit media use to extremely low levels may not be replacing that time with other enriching activities. It may be more reasonable for interventions and recommendations to focus on shifting the quality of children’s media use by increasing educational content, rather than decreasing the overall quantity, at least for children who receive moderate levels of media use. Importantly, these data do show a negative relation between high levels of media use and children’s language gains. For these children, interventions to decrease media use and replace it with more enriching activities could be warranted. This distinction highlights the value of conducting screening and differentiating recommendations to families based on their existing media use.

Our findings also suggest that joint media engagement may play an important buffering role in the relation between media use and children’s early literacy skills, in line with recent research (Hutton et al., 2020). The American Academy of Pediatrics recommendations include a focus on co-using media with children (AAP Council on Communications and Media, 2016) and other research has shown that co-use can enhance positive effects (e.g., Strouse et al., 2013) and buffer negative effects of media (Nathanson et al., 2002). These findings support this recommendation and suggest that joint media engagement may be helpful for limiting negative effects of media use on children’s developing literacy skills.

Despite its strengths, there are several limitations to this study. First, both language and literacy are complex constructs and were measured here through single standardized measures. Future research should expand the measures that are used to more comprehensively understand the relation between media and multiple facets of language and literacy development. Furthermore, media use was reported by parents, whose responses may be limited by memory challenges and/or social desirability, as is common in this literature (see Madigan et al., 2020). Future studies would do well to include more objective measures of children’s media exposure, such as ecological momentary assessment or passive device tracking. Additionally, our measure of joint media engagement was self-reported by caregivers and asked about general approaches to media use in the home rather than specific instances of joint media engagement. We took this approach because we expected that instances of joint media engagement might be relatively rare and hard to capture. However, it would be beneficial for additional research to use alternative methods of assessing joint media engagement, such as observation. Notably, although we examined gains across a school year to avoid some of the limitations of studies using only one time point, these relations are still correlational and do not justify causal conclusions. Rigorous correlational research using multiple time points can justify possible targets for interventions, which could then provide causal evidence for the relations between these variables.

The current study makes several important contributions to the literature in this area. By accounting for non-linear relations and taking into consideration the characteristics of media use, the current results begin to provide a more nuanced understanding of the relation between media use and language and literacy development. Our results demonstrate the importance of going beyond linear associations and understanding possible buffers of the role of children’s media use on child development.

## Data Availability Statement

The raw data supporting the conclusions of this article will be made available by the authors upon request, without undue reservation.

## Ethics Statement

The studies involving human participants were reviewed and approved by The Ohio State University Institutional Review Board. Written informed consent to participate in this study was provided by the participants’ legal guardian/next of kin.

## Author Contributions

LJ, KP, T-JL, and JL contributed to conceptualization of the larger project and obtained funding. RD and JL contributed to the conceptualization of the current study and conducted data analyses. RD drafted the work. JL, LJ, KP, and T-JL contributed to editing and revising and gave final approval of the version to be submitted.

### Conflict of Interest

The authors declare that the research was conducted in the absence of any commercial or financial relationships that could be construed as a potential conflict of interest.

## Footnotes

^1^Seven children were retained in kindergarten during the second year of testing.

^2^In line with studies showing that low-SES and minority families are underrepresented in research (Myers et al., 1992; Gross et al., 2001), this sub-sample had higher maternal education (Ï‡2 = 42.8, *p* < 0.0001), fewer single-adult homes (Ï‡2 = 13.1, *p* = 0.01), and more White families than the full sample (*Ï‡2* = 52.3, *p* < 0.0001), suggesting that even when these families consented to being part of the study they were less likely to return the survey and/or respond to survey items.
